# Diffusion and physical constraints limit oxidative capacity, capillary supply and size of muscle fibres in mice and humans

**DOI:** 10.1113/EP092750

**Published:** 2025-06-07

**Authors:** Hans Degens, Guy A. M. Messa, Jason Tallis, Alessandra Bosutti, Tomas Venckunas, Ismail Adeniran, Rob C. I. Wüst, Paul W. Hendrickse

**Affiliations:** ^1^ Department of Life Sciences, Institute of Sport Manchester Metropolitan University Manchester UK; ^2^ Institute of Sport Science and Innovations Lithuanian Sports University Kaunas Lithuania; ^3^ Higher Institute of Medical Technology ISTM‐Kinshasa Kinshasa Democratic Republic of Congo; ^4^ Faculty of Medicine University Kasa‐Vubu (UKV) Boma Democratic Republic of Congo; ^5^ Faculty of Medicine University de Bandundu (UNIBAND) Bandundu Democratic Republic of Congo; ^6^ Center for Physical Activity Sport and Exercise Science, Richard Crossman Building Coventry University Coventry UK; ^7^ Department of Life Sciences University of Trieste Trieste Italy; ^8^ Centre for Advanced Computational Science Manchester Metropolitan University Manchester UK; ^9^ Department of Human Movement Sciences, Faculty of Behavioural and Movement Sciences Vrije Universiteit Amsterdam, Amsterdam Movement Sciences Amsterdam The Netherlands; ^10^ Lancaster Medical School Lancaster University Lancaster UK

**Keywords:** capillarisation, capillary domain, microvasculature, muscle fibre, oxidative capacity

## Abstract

It has been suggested that angiogenesis during skeletal muscle fibre hypertrophy allows escape from the ‘size constraint’, which is the inverse relationship between oxidative capacity and muscle fibre cross‐sectional area (FCSA). It is, however, not known whether there are any limitations to the combinations of FCSA, oxidative capacity and capillary supply to an individual fibre. We determined the FCSA, oxidative capacity and capillary supply to fibres from highly resistance‐trained men before and after superimposed endurance training, recreationally active men and women, and different mouse muscles. Both the oxidative capacity and the number of capillaries around a fibre (CAF) per FCSA (CAF/FCSA) showed an upper limit at each FCSA, irrespective of species, muscle origin or training status. The upper limit of fibre oxidative capacity was likely determined by diffusion constraints. The upper limit of CAF/FCSA was determined by physical constraints where (i) there is no further reduction in maximal diffusion distance to the core of a fibre beyond a CAF of 2, and (ii) the reduction in fibre area supplied by a capillary diminishes exponentially with an increase in CAF. The calculated upper limits of oxidative capacity and CAF/FCSA of a fibre of a given FCSA were linearly related. Irrespective of species, sex, muscle of origin and training status, our data indicate that diffusion limitations and physical limitations to capillary placement around a fibre place an upper limit on the oxidative capacity and capillary supply to a fibre of a given size, respectively.

## Abstract (highlights)

- **What is the central question of this study?**
  For skeletal muscles, are there are any limitations to the combinations of fibre cross‐sectional area, oxidative capacity and capillary supply to an individual fibre?
- **What is the main finding and its importance?**
  Diffusion constraints limit the oxidative capacity of a fibre of a given size. Physical constraints to capillary placement around a fibre and diminishing returns for additional capillaries limit the capillary supply to a fibre of a given size. These constraints are independent of species, sex, muscle and training status.

## 1 INTRODUCTION

Capillaries are the smallest vessels of the cardiovascular system and are not only essential for oxygen and carbon dioxide exchange between the blood and muscle fibres, but are also important for dissipation of heat and metabolites, and the delivery of nutrients and hormones (Hendrickse & Degens, 2019). As it is thought that the oxygen flux from the capillary to the mitochondria is driven by a diffusion gradient (Hill, 1965; Krogh, 1919), the size of a muscle fibre and its oxidative capacity may be limited by diffusion limitations (Degens, 2012). In line with this hypothesis is the inverse relationship between the oxidative capacity and the size of individual fibres across muscles and species (Van der Laarse et al., 1998). This inverse relationship (referred to here as the ‘size constraint’) was suggested to reflect oxygen diffusion limitations to the size and oxidative capacity of a fibre to prevent the development of anoxic cores (Van der Laarse et al., 1998).

However, the largest drop in oxygen pressure ($P_{O_{2}}$) occurs between the erythrocyte and the sarcolemma (Poole & Musch, 2023) leaving a small $P_{O_{2}}$ gradient within the muscle cell as suggested by the shallow oxymyoglobin (MbO_2_) gradient in working dog m. gracilis muscle fibres (Honig et al., 1997; Poole & Musch, 2023). It should be noted, however, that substantial $P_{O_{2}}$ gradients can exist in the presence of shallow oxymyoglobin (MbO_2_) gradients (Degens et al., 2024) due to the shape of the myoglobin (Mb) dissociation curve. In addition, metabolic processes are typically not greatly, if at all, limited by diffusion (Kinsey et al., 2011). This and the absence of an elevated oxygen diffusion capacity after immobilisation despite reduced diffusion distances (Hepple et al., 2000) challenge the idea that diffusion limitations underlie the ‘size constraint’. Such an absence of diffusion constraints is perhaps realised by the reticular mitochondrial ‘power grid’, where mitochondria close to capillaries create the proton gradient that in the core can be used to generate ATP with reduced need of oxygen (Clanton, 2019; Glancy et al., 2015; Parry et al., 2024). This then perhaps explains the enormous variation in local maximal oxygen consumption supported per capillary (Bosutti et al., 2015). Furthermore, there have been several cases reported where the ‘size constraint’ seems to have been broken, as muscle hypertrophy in mice (Ballak et al., 2016; Hendrickse et al., 2020; Omairi et al., 2016), rats (Frischknecht & Vrbova, 1991) and humans (Hendrickse, Venckunas et al., 2021) resulted in a higher oxidative capacity for a fibre of a given size than seen in the non‐hypertrophied muscles. Perhaps even more striking are the observations that hypertrophy was not attenuated, nor muscle size diminished, when the hypertrophic stimulus was combined with endurance training (Hendrickse, Krusnauskas et al., 2021; Hendrickse, Venckunas et al., 2021) or chronic electrical stimulation (Frischknecht & Vrbova, 1991).

Even though oxygen diffusion gradients are rather small, a diffusion constraint on fibre size and oxidative capacity cannot be excluded, as metabolites, heat and waste products must be removed from the working muscle. Indeed, in the animal kingdom, adult skeletal muscle fibres may work at ‘the brink of diffusion limitation’, and exceptional enlargement of fibres is accompanied by redistribution of mitochondria to the periphery, metabolic compartmentalisation and/or splitting of fibres (Kinsey et al., 2011). In addition, we found that the hypertrophy and concomitant elevated oxidative capacity were at least partly attributable to angiogenesis (Ballak et al., 2016; Hendrickse, Krusnauskas et al., 2021) that may have reduced the diffusion distances from the capillary to the mitochondria in the core of the fibre, and increased the capillary contact area with the muscle fibres (Parry et al., 2024). This suggestion was further supported by the observations that: (i) blunted hypertrophy in old mice was accompanied by attenuated angiogenesis (Ballak et al., 2016; Hendrickse, Krusnauskas et al., 2021), (ii) the similar time course of angiogenesis and hypertrophy (Plyley et al., 1998), and (iii) the positive relationship between muscle capillarisation and amount of hypertrophy in older people (Moro et al., 2019; Snijders et al., 2017). Even so, during maturational muscle growth, angiogenesis is proportionally less than the increase in fibre size, resulting in a decrease in capillary density (Degens et al., 2006; Ripoll et al., 1979). In fact, an inverse relationship between average fibre size and muscle capillary density has been seen across species and muscles (Hudlicka, 1984). Although all these observations were done at the level of the whole muscle, they are qualitatively similar to the inverse relationship between fibre size and oxidative capacity seen in individual fibres (Van der Laarse et al., 1998). We therefore hypothesise that there are limits to the oxidative capacity, local capillary supply and cross‐sectional area of individual muscle fibres. The objectives of the present study were to (i) assess any limitations to the possible combinations of the size, oxidative capacity and capillary supply to a fibre, and in case there are limits (ii) consider factors that may underlie these limited combinations at the level of individual muscle fibres.

To investigate this, we assessed these relationships in (i) mouse muscle fibres, which are typically half the size of human fibres yet with a higher oxidative capacity, (ii) fibres from recreationally active people, and (iii) fibres from highly resistance‐trained men, who have much larger fibres than untrained people, before and after a superimposed endurance training programme, to cover a wide range of fibre sizes and fibre oxidative capacity. It is important to emphasise that determination of the limits of size, oxidative capacity and capillary supply to a fibre requires the assessment of these parameters in individual fibres, and that this cannot be derived from average values.

## 2 METHODS

This study used histological sections from pre‐bed‐rest biopsies. The capillary supply, size, type and oxidative capacity of individual fibres were determined in the m. soleus (SOL; 1326 fibres) and m. vastus lateralis (VL; 1524 fibres) from 13 men and 6 women (healthy recreationally active; 23–54 years). The biopsies were taken during the AGBRESA study. The details of the methods, participant characteristics and average morphology data have been described previously (Hendrickse et al., 2022). In that study, we found no significant differences in muscle morphology between men and women, and we therefore pooled the fibres from both sexes. The Ethics Committee of the North Rhine Medical Association (reference no. 2018143) in Düsseldorf, Germany, approved the study and it was included in the German Clinical Trials Register (DRKS‐ID: DRKS00015677).

In addition, 2418 muscle fibres from the VL from fifteen 23‐ to 77‐year‐old highly resistance‐trained men (competitive and retired bodybuilders and men who resistance trained recreationally for a minimum of 5 years) were analysed. The fibres were from biopsies taken before (*n* = 1103) and after (*n* = 1315) a 10‐week endurance training programme superimposed on their regular training that resulted in angiogenesis and an increase in oxidative capacity without muscle fibre atrophy (Hendrickse, Venckunas et al., 2021). The fibres from the highly resistance‐trained men in this study are comparable in size to those of elite bodybuilders (MacDougall et al., 1984), who are thought to exhibit the limits of skeletal muscle hypertrophy within humans. More details of these highly resistance‐trained men are presented elsewhere (Hendrickse, Venckunas et al., 2021). The Kaunas Regional Biomedical Research Ethics Committee (Authorisation number BE‐10‐4) approved the study.

All participants gave written informed consent prior to providing biopsies and the studies adhered to the standards of the *Declaration of Helsinki*.

Young‐adult female CD‐1 mouse SOL (*n* = 6; 1565 fibres), diaphragm (DIA; *n* = 7; 2054 fibres) and extensor digitorum longus (EDL; *n* = 3; 478 fibres) muscles were the same as those used in Messa et al. (2019). The animals received food and water ad libitum and were kept at a 12 h light–dark cycle at 22°C. They were sacrificed at the age of 20 weeks by cervical dislocation in accordance with the British Home Office Animal (ScientificProcedures) Act 1986, Schedule 1 and the study adhered to the ARRIVE guidelines (Percie du Sert et al., 2020). The details of the methods, body and muscle masses are described in Messa et al. (2019).

### 2.1 Capillary and succinate dehydrogenase staining

The previous human (Hendrickse, Venckunas et al., 2021; Hendrickse et al., 2022) and mouse (Messa et al., 2019) studies show examples of the staining used. Those studies also present the average data of the morphological parameters assessed here, but did not address the relationship between the different morphological parameters in individual fibres.

Serial sections (10 µm) were cut in a cryostat (CM3050S; Leica, Nußloch, Germany) and stained for capillaries, and succinate dehydrogenase (SDH) as a marker for fibre oxidative capacity. In mouse muscles, capillaries were identified by incubation with biotinylated *Griffonia* (*Bandeira*) *simplicifolia* lectin (GSL I; Vector Laboratories, Peterborough, UK; 50 µg mL^−1^ diluted in 1% BSA/HEPES) (Messa et al., 2019) and in human muscle with *Ulex europaeus* Agglutinin I fluorescein (1:250) (Thermo Fisher Scientific, Waltham, MA, USA) (Hendrickse, Venckunas et al., 2021; Hendrickse et al., 2022). Serial sections were stained for SDH as described previously (Hendrickse, Venckunas et al., 2021; Hendrickse et al., 2022; Messa et al., 2019) and photos taken on a light microscope with a 660‐nm interference filter and a black and white AxioCam ICMI camera (Zeiss, Oberkochen, Germany). Images were analysed using ImageJ (National Institutes of Health, Bethesda, MD, USA). To measure the optical density (OD) of a given fibre, the outline of the fibre was drawn, and the background OD subtracted. For each section, a separate calibration curve was made with filters of known OD. The calibration curve was used to convert the absorbance values of the SDH staining into OD values. It has been shown in healthy single muscle cells from *Xenopus* (van der Laarse et al., 1989), rat (Van der Laarse et al., 1998) and humans (Bekedam et al., 2003) that the OD of the SDH staining at 660 nm is linearly related to the maximal oxygen consumption per volume of a fibre, referred to here as the mass‐specific SDH.

### 2.2 Capillarisation

**FIGURE 1:**
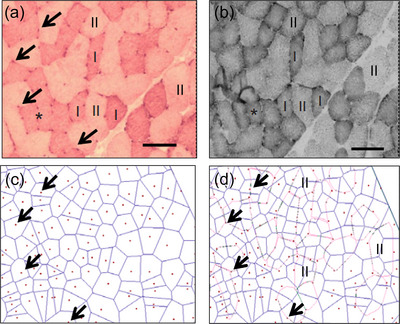
Stained skeletal muscle sections and corresponding capillary domains. (a) Section stained for type I fibres (dark; type II fibres are lighter) and capillaries (indicated with arrows). (b) A serial section stained for succinate dehydrogenase. (c) Capillary domains (blue borders with red capillary). (d) capillary domains overlaid with type II outlines (pink). Scale bar 100 µm. Arrows indicate corresponding capillaries in (a, c, d). Corresponding fibres in (a, b, d) indicated by *, I and II, with image d only showing type II fibres (II). Adapted from Bosutti et al. (2015).

The capillary supply to individual fibres was assessed by the method of capillary domains (Figure 1). A capillary domain is defined as the area surrounding a capillary delineated by equidistant boundaries from adjacent capillaries (Hoofd et al., 1985). The capillary domain appears to be a good reflection of the capillary oxygen supply area (Al‐Shammari et al., 2014) and is illustrated in Figure 1c. Capillary coordinates and the coordinates of fibre outlines were collected with BTablet and analysed with AnaTis (https://hoofd.info/louis/s/s_Apps.html) for mouse muscles and muscles from highly resistance‐trained men, and collected and analysed with DTect (https://ora.ox.ac.uk/objects/uuid:6d128833‐4c00‐46bd‐b7aa‐10145e5091b9) for the AGBRESA human muscles.

The method of capillary domains allows one to not only obtain domain areas (hereafter referred to as ‘domains’) and indices of overall muscle capillarisation, but also muscle fibre‐specific indices of capillary supply as described previously (Bosutti et al., 2015; Degens et al., 1992). In Figure 1d multiple capillary domains (blue) overlap each muscle fibre (pink). The number of domains overlapping a fibre is similar, but not entirely identical (i.e. the rare instance where a fibre misses direct contact with a capillary) to capillaries around a fibre, and to help understanding we present it here as capillaries around a fibre (CAF). We also calculated the local capillary‐to‐fibre ratio (LCFR), defined as the sum of the domain fractions overlapping a fibre (see Figure 4a). LCFR thus considers that a capillary typically supplies more than one fibre. The capillary fibre density (CFD) is calculated as the LCFR divided by the fibre cross‐sectional area (FCSA). Another measure of capillary density of a fibre was CAF/FCSA. The LCFR and CFD were not available for the highly resistance‐trained men. In addition to these parameters, AnaTis provides the perimeter of a fibre, that was not available for the highly resistance‐trained men as these samples were analysed with Dtect.

### 2.3 Statistical analyses

As the data were not normally distributed, differences between muscles and groups were tested with the non‐parametric Kruskall–Wallis test. Pearson correlations between parameters were calculated. Differences and correlations were considered significant at *P *< 0.05.

## 3 RESULTS AND DISCUSSION

As capillarisation is not only important for oxygen delivery, but also for delivery of substrates and removal of heat and metabolic waste products, we start here with assessing the relationship between the size (FCSA) and the capillary fibre density (CFD). Of the highly resistance‐trained men, 13 fibres were larger than 25,000 µm^2^ (the largest fibre was 38,000 µm^2^ and all with a normal form factor, indicating that the large size is not due to a longitudinal rather than cross‐sectional profile) but they did fit the patterns described below. While rare, fibres larger than 25,000 µm^2^ have been found occasionally in muscle biopsies from bodybuilders (Meijer et al., 2015). Mean FCSA for these men (Hendrickse, Venckunas et al., 2021) was comparable to those found in other studies of bodybuilders and powerlifters (MacDougall et al., 1984). To not unnecessarily expand the scales in the graphs, these fibres are not shown but were included in any curve fitting.

### 3.1 Capillary fibre density and fibre size

**FIGURE 2:**
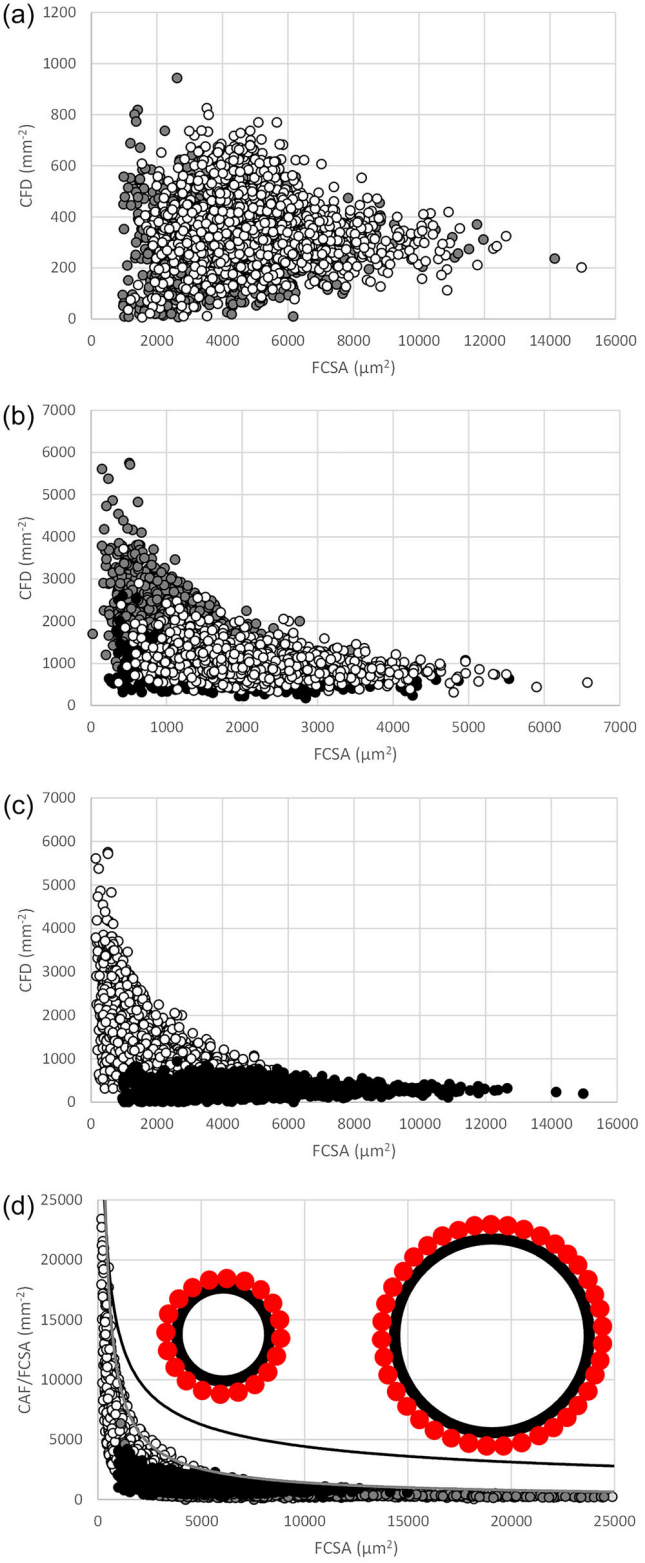
Number of capillaries per mm^2^ of an individual muscle fibre (capillary fibre density; CFD) versus fibre cross‐sectional area (FCSA) in (a) human m. soleus (white circles) and m. vastus lateralis (grey circles), (b) mouse m. soleus (white circles), m. diaphragmaticus (grey circles) and m. extensor digitorum longus (black circles), and for (c) CFD and (d) capillaries around a fibre per FCSA (CAF/FCSA) in mouse (white circles) and human (grey and black circles) muscle fibres combined, including fibres from highly resistance‐trained men. Each data point represents an individual muscle fibre. The black line in (d) reflects the maximum possible value of CAF/FCSA assuming a circular circumference and a capillary diameter of 8 µm at each FCSA. White circles, mouse; black circles, recreationally active human; highly resistance‐trained men before (dark grey circles) and (light grey circles) after 10 weeks superimposed endurance training (note, the overlap with pre‐ is so large that only at the right end a data point for post training be seen, while *n* = 1315 fibres post). The grey line indicates the curve fitted to the highest CAF/FCSA for any FCSA (CAF/FCSA = 3,621,508 × FCSA^−0.85166676^). The inset in (d) illustrates a theoretical ceiling for fibre capillarisation; if the perimeter of a muscle fibre (large black circles) is completely covered with capillaries (small red circles) the CAF/FCSA for that FCSA is maximised, as illustrated with two fibres, one with half the radius of the other.

Figure 2 shows the relationship between CFD and FCSA. In human muscle (Figure 2a) there was no significant relationship in the VL and a weak correlation in the SOL (*R* ^2^ = 0.008; *P* = 0.001), even though the proportion of type I fibres was ∼65% in the SOL and ∼36% in the VL (Hendrickse et al., 2022). This thus corresponds with previous observations that fibre type has little, if any, impact on the capillary supply to a fibre (Ahmed et al., 1997; Barnouin et al., 2017; Wüst, Gibbings et al., 2009). In mouse muscles, the DIA had the smallest fibres and a higher CFD than the SOL and EDL (*P *< 0.001; Figure 2b).

The most remarkable observation is, however, that in human, and even more strikingly in mouse muscles, there is a clear‐cut upper limit of possible combinations of CFD and FCSA, where the CFD at a given FCSA does not exceed a maximum value and vice versa. As humans have larger fibres than mice, we next sought to see whether there was any overlap or continuation of this upper limit when human and mouse muscle fibres were combined. This was indeed the case (Figure 2c) and indicates that there is an upper limit of the FCSA for a given CFD (and vice versa) across different muscles and across species.

While an inverse curvilinear relationship between muscle capillary density and average FCSA has been reported between different muscles, muscles from different species (Hudlicka, 1984) and in growing rat skeletal muscle (Ripoll et al., 1979), this has not been systematically evaluated at the level of the individual fibre. This is the first time an upper limit for CFD and FCSA combinations at the individual fibre level is reported. But what is the cause of this limit to possible combinations between CFD and FCSA? Is it perhaps indicative of a diffusion constraint for FCSA at a given CFD and/or a physical limitation of the number of capillaries that can be placed around a fibre?

### 3.2 Local capillary‐to‐fibre ratio and fibre size

**FIGURE 3:**
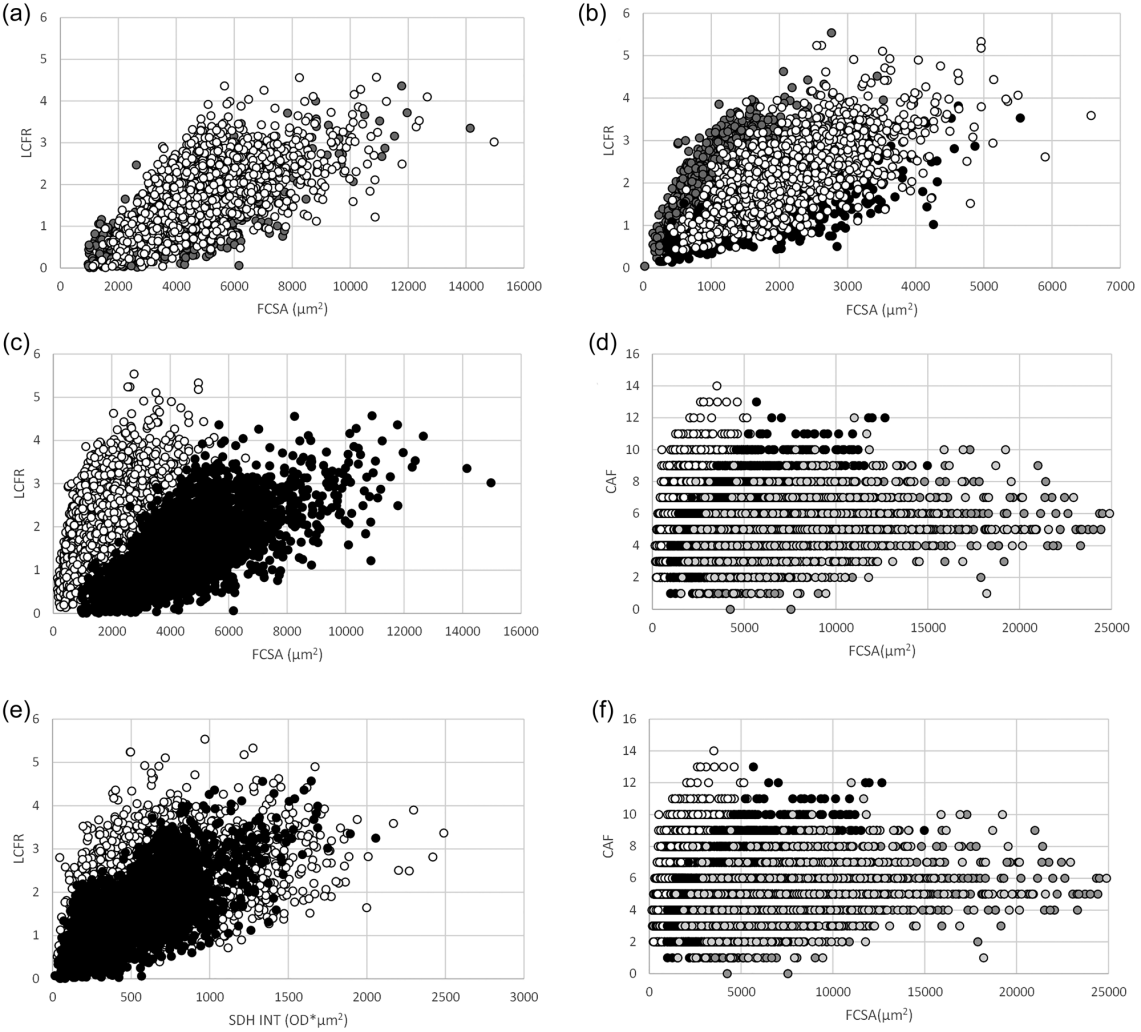
Number of capillaries supplying an individual fibre (local capillary‐to‐fibre ratio; LCFR) versus fibre cross‐sectional area (FCSA) in recreationally active (a) human m. soleus (SOL) (white circles) and m. vastus lateralis (VL) (grey circles), (b) mouse m. soleus (SOL) (white circles), m. diaphragmaticus (DIA) (grey circles) and m. extensor digitorum longus (EDL) (black circles) and (c) LCFR and (d) capillaries around a fibre (CAF), and (e) LCFR or (f) CAF versus integrated succinate dehydrogenase optical density (SDH INT) in mouse SOL, EDL and DIA (white circles) and recreationally active human SOL and VL (black circles) muscle fibres combined. (d, f) include VL fibres from highly resistance‐trained men. White circles, SOL, EDL and DIA from mouse; Black circles, SOL and VL fibres from recreationally active human; VL from highly resistance‐trained men before (dark grey circles) and after (light grey circles) 10 weeks superimposed endurance training. Each data point represents an individual muscle fibre.

To start to answer this question, we first removed the contribution of the FCSA to CFD, by plotting the LFCR against FCSA. There was a positive relationship between the LCFR and the FCSA that was similar in the SOL and VL in human muscles (slope in µm^−2^) 0.0003; *R* ^2^ = 0.42 and 0.50 for SOL and VL, respectively; both *P *< 0.001, Figure 3a). In mouse muscles the slope was steepest for the DIA, least steep in the EDL (*P *< 0.001), with the slope of the SOL in between (DIA: slope 0.0011, *R* ^2^ = 0.48; SOL: slope 0.0007, *R* ^2^ = 0.49 and EDL: slope 0.0005, *R* ^2^ = 0.56; all *P *< 0.001; differences between slopes *P *< 0.001; Figure 3b). This relationship was steeper in mouse than human muscle fibres (*P *< 0.001, Figure 3c). The positive relationship between LCFR and FCSA is in line with previous observations that fibre size is an important determinant of the capillary supply to a fibre in normal (Ahmed et al., 1997; Bosutti et al., 2015; Wüst, Gibbings et al., 2009) and hypertrophied (Degens et al., 1994) muscles, across muscle regions with different oxidative capacity (Degens et al., 1994; Kissane et al., 2018), muscles from rats with spinal cord injury (Kissane et al., 2018), old rats (Degens et al., 1994), older people (Barnouin et al., 2017) and fish (Egginton et al., 1988). Although the slopes of the relationships differ, the observations of these studies and the present study indicate that the relationship holds across muscles, species and conditions, such as ageing, hypertrophy and spinal cord injury. The question arises, however, of what underlies the different slopes in different conditions, and relevant here, what explains that this relationship is steeper in mouse than in human muscle.

### 3.3 Considerations of the local capillary‐to‐fibre ratio and capillaries around a fibre

**FIGURE 4:**
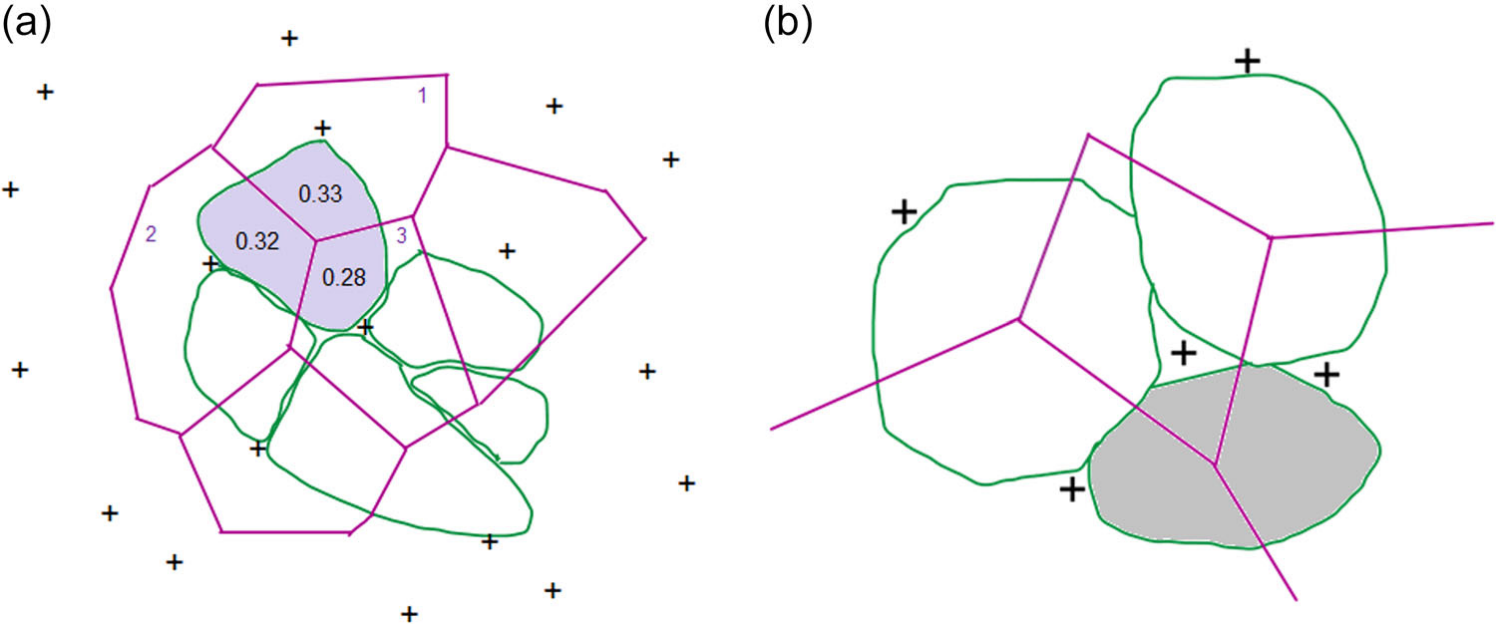
Illustration of the method of capillary domains. (a)The relationship between domains (shown as purple polygons) and fibres (shown as green shapes). Each capillary (shown as crosses) supplies multiple fibres (Adapted from Egginton et al., 1988). The local capillary‐to‐fibre ratio (LCFR) is the sum of the domain fractions which supply a given fibre. Here capillary 1 supplies 0.33 of its domain (of 1.0) to a fibre while capillaries 2 and 3 provide 0.32 and 0.28, and the LCFR for that particular fibre would be 0.93 (0.33 + 0.32 + 0.28 = 0.93). (b) A visual representation of the relationship between the number of capillaries around a fibre (CAF) and the number of capillaries supplying an individual fibre (LCFR). In all cases the number of CAF is 3, primarily because of the three capillaries around the small (grey) fibre. However, the local capillary‐to‐fibre ratio (LCFR) for the grey fibre will be lower than those of the larger fibres as most of the domain areas cover the larger fibres. Hence the fraction of the domains overlapping a fibre, and sum of the fractions of the domains, is larger for these larger fibres. This problem will affect a small fibre surrounded by large fibres (as in human muscle), but not a small fibre surrounded by small fibres (as in the mouse muscles), where the fractions of overlap will not be ‘taken away’ from the small fibres.

In Figure 4b, all fibres are surrounded by three capillaries and hence have a CAF of 3, which is primarily because of the three capillaries around the small (grey) fibre. The LCFR for the grey fibre will be lower than those of the larger fibres as larger fractions of the domain areas cover the larger fibres, and hence the sum of the fractions of the domains overlapping a fibre, the LCFR, is larger for the larger fibres than the small fibre. This problem will affect a small fibre surrounded by large fibres (as in human muscle), but not a small fibre surrounded by small fibres (as in the mouse muscles), where the fractions of overlap will not be ‘taken away’ from the small fibres, and hence may explain the steeper relationship between LCFR and FCSA in mouse than human muscle (*P *< 0.001). The CAF is not affected by this phenomenon, but still shows a steeper relationship with FCSA in mouse than human muscles (*P *< 0.001, Figure 3d). Furthermore, like the CFD versus FCSA relationship (Figure 2c), the CAF/FCSA, indicating the number of capillaries per unit fibre area, has a sharply delineated upper limit for a given FCSA (Figure 2d). CAF and LCFR thus give qualitatively similar results and hence the ‘taking away’ of domain fractions from small fibres by large fibres does not explain the steeper LCFR or CAF versus FCSA slope in mice than human fibres. What then is the explanation for the steeper slope in mouse than in human muscle?

### 3.4 Diffusion constraint of oxidative capacity and fibre size

One potential contributor to the steeper slope of the LCFR or CAF versus FCSA in mice than human muscle is the oxidative capacity of the muscle fibres. This has been seen most clearly in fish, where fast glycolytic fibres show a less steep relationship between LCFR (or CAF) and FCSA than slow oxidative fibres (Egginton et al., 1988), and within rat muscles (e.g. the plantaris and tibialis anterior muscles), where the relationship was steeper in the oxidative than the glycolytic region (Degens et al., 1994; Kissane et al., 2018).

**FIGURE 5:**
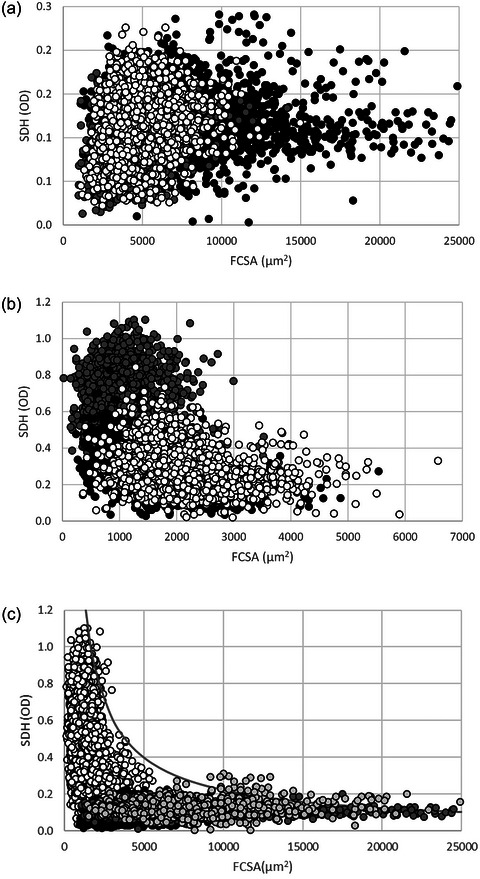
The oxidative capacity of an individual fibre (measured as succinate dehydrogenase optical density (SDH OD) versus fibre cross‐sectional area (FCSA) in (a) recreationally active human m. soleus (SOL) (white circles) and m. vastus lateralis (VL) (grey circles), and VL from highly resistance‐trained men (black circles), (b) mouse SOL (white circles), m. diaphragmaticus (DIA) (grey circles) and m. extensor digitorum longus (EDL) (black circles) and (c) SOL, EDL and DIA fibres from mice (white circles), SOL and VL fibres from recreationally active humans (black circles) and VL from highly resistance‐trained men before (dark grey circles) and after (light grey ciclces) 10 weeks’ superimposed endurance training combined. Each data point represents an individual muscle fibre. The grey line indicates the curve fitted to the highest SDH (OD) versus FCSA combinations (SDH (OD) = 539 × FCSA^−0.84605321^).

We therefore also determined to what extent the SDH OD, a measure of the oxidative capacity of a fibre, differed between fibres from different mouse muscles, and between mouse and human muscle fibres. We found that the SDH OD in the fibres from recreationally active people was higher in fibres from the SOL than the VL (*P *< 0.001; Figure 5a). Among the mouse muscles, fibres from the DIA had a higher SDH OD than the SOL, and that from the SOL was higher than the SDH OD of EDL fibres (*P *< 0.001; Figure 5b). Mouse muscle fibres had a higher SDH OD than human muscle fibres, but human fibres were larger than mouse muscle fibres (*P *< 0.001; Figure 5c). Despite an enormous variability in the SDH OD for a given FCSA, the striking observation in Figure 5c – where data from recreationally active and highly resistance‐ trained humans before and after superimposed endurance training were combined with the mouse data – is that there appears to be a limit to the combinations of FCSA and SDH OD where the SDH OD does not exceed a certain ceiling at a given FCSA and vice versa. This ‘ceiling’ resembles the reported inverse relationship between FCSA and maximal oxygen consumption of a fibre (Van der Laarse et al., 1998) that was suggested (i) to be the consequence of oxygen diffusion constraints that limit the size of a fibre with a given oxidative capacity and (ii) to reflect therefore a trade‐off between the aerobic capacity and size of a muscle fibre. At first glance both the higher SDH OD and FCSA from the VL of highly resistance‐trained men pre‐ and post‐superimposed endurance training than those from the VL of recreationally active men and women (*P *< 0.001) suggests that the fibres of the resistance‐trained men break this ceiling. However, even the largest fibres appear to obey this ‘ceiling’ of FCSA and SDH OD combinations (Figure 5c).

### 3.5 Total fibre oxidative capacity and fibre capillarisation

To address the question of whether the different slopes between LCFR (or CAF) and FCSA between mouse and human muscle fibres are explicable by differences in the oxidative capacity of a fibre, we assessed the relationship between the maximal oxygen demand and the capillary supply of each fibre. Knowing the mass‐specific oxidative capacity (SDH OD) and the FCSA of a fibre we can calculate the total oxidative capacity (or maximal oxygen demand) of a fibre as FCSA × SDH OD (the integrated SDH OD for a fibre: SDH INT). Here we show for the first time that the slopes of the relationships between LCFR (Figure 3e) or CAF (Figure 3f) with SDH INT (all *P *< 0.001) are qualitatively similar in mice and humans, indicating that the capillary supply to a fibre is positively related to the total oxidative capacity (maximal oxygen demand) of a fibre. In the highly resistance trained men, there was, surprisingly, a negative relationship between CAF and SDH INT, but with a rather shallow slope, as can be seen in Figure 3f (*P *< 0.001). However, it is clear from Figure 3c and d that the FCSA was the main determinant of the LCFR and CAF, respectively. The difference in slope between men and mice reflects the contribution of the oxidative capacity in the determination of the capillary supply to a fibre. This is in line with our previous observations that the FCSA was the prime determinant of the capillary supply to a fibre with only a minor impact of the oxidative capacity of the muscle fibre (Barnouin et al., 2017; Bosutti et al., 2015; Wüst, Jaspers et al., 2009).

### 3.6 Capillary supply per perimeter

**FIGURE 6:**
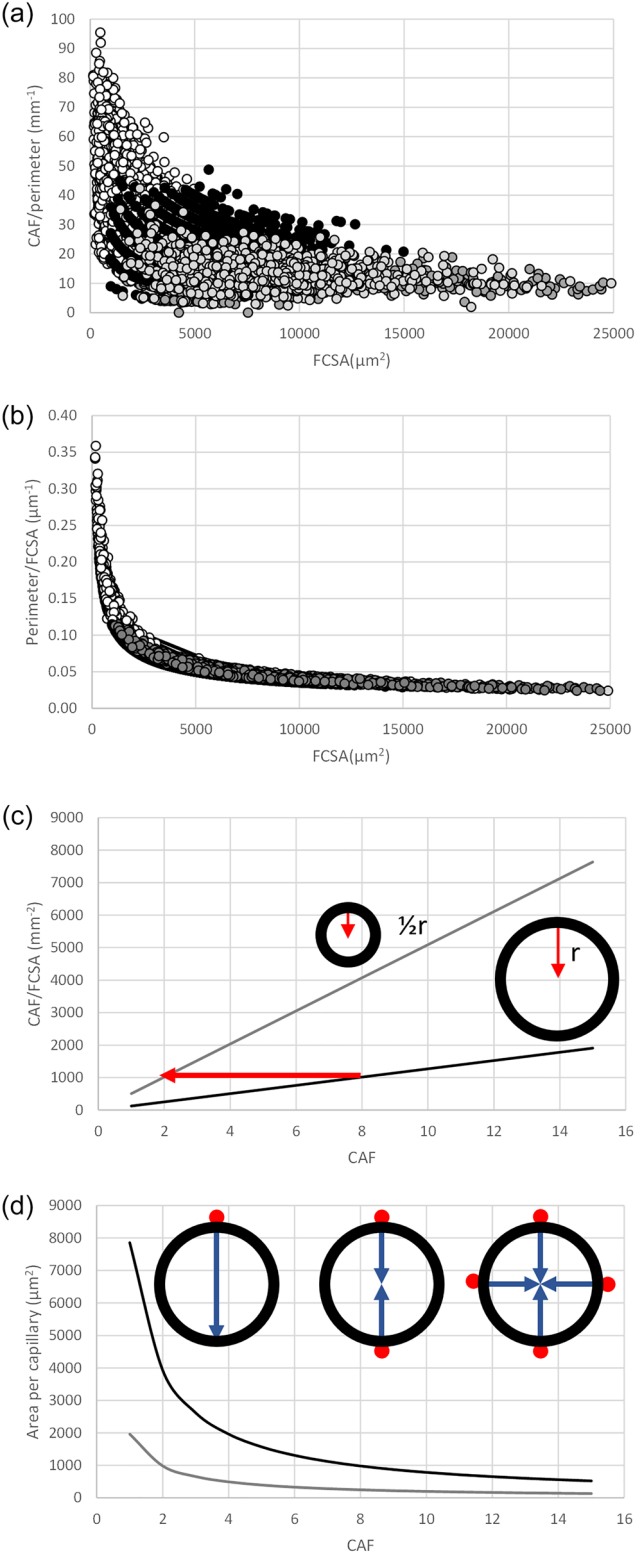
Impact of number of capillaries around a fibre on difussion distance and muscle fibre area supplied by a capillary. (a) Capillaries around a fibre (CAF) per fibre perimeter versus fibre cross‐sectional area (FCSA) in muscle fibres from m. soleus (SOL), m. diaphragmaticus (DIA) and m. extensor digitorum longus (EDL) from mice (white circles), SOL and m. vastus lateralis (VL) from recreationally active human (black circles) and the VL from highly resistance‐trained men before (dark grey circles) and (light grey circles) after 10 weeks superimposed endurance training. (b) The perimeter per FCSA assuming a circular circumference in human fibres, and the actual perimeter in muscle fibres from recreationally active human soleus and VL (white circles) and the VL from highly resistance‐trained men before (dark grey circles) and (light grey circles) after 10 weeks superimposed endurance training. Each data point represents an individual muscle fibre. (c) the number of capillaries around a fibre per fibre cross‐sectional area (CAF/FCSA) versus CAF in a fibre with a radius of 50 (black line) and 25 µm (grey line), illustrated by the cells with a radius of 1 and ½r. The red arrow indicates that 4× as many capillaries are needed around the large (8) than the small (2) fibre to have the same CAF/FCSA. (d) The decrease in the area of a fibre supplied by a capillary. Fibre radius 50 µm (black line) and 25 µm (grey line). The inset in (d) shows that increasing CAF from 1 to 2 halves the maximal diffusion distance (indicated by arrows), while the maximal diffusion distance does not decrease further with further increases in CAF.

The capillary contact area per fibre perimeter may be a better reflection of the oxygen exchange capability between capillaries and muscle fibres (Hepple et al., 2000, 1997), as the greatest drop in oxygen pressure occurs between the red blood cell and the sarcolemma as suggested by the shallow MbO_2_ gradient within working myofibres (Honig et al., 1997; Poole & Musch, 2023). It should be noted, however, that due to the shape of the Mb dissociation curve there may a substantial $P_{O_{2}}$ gradient even if the MbO_2_ gradient is shallow (Degens et al., 2024). We therefore also calculated the CAF and LCFR per perimeter, assuming a circular circumference for human muscle fibres, and using the measured perimeter for the fibres from the mice and the highly resistance‐trained men (Figure 6a). The upper limit is qualitatively similar to that for the CFD (Figure 2a–c) or CAF/FCSA (Figure 2d) versus FCSA relationship. But what could underlie the apparent threshold, not only for CFD or CAF/FCSA, but also for the CAF or LCFR (data not shown) per perimeter, for a fibre with a given FCSA?

### 3.7 Physical constraints of capillary placement

Although capillaries may be found inside a muscle fibre in invertebrates, such as the blue crab (*Callinectes sapidus*) with fibres >600 µm in diameter (Hardy et al., 2009), in vertebrate muscles capillaries are only located at the periphery of a muscle fibre. Figure 6b illustrates that with increasing FCSA, the perimeter : FCSA ratio decreases. Particularly fibres smaller than 2500 µm^2^ have a large perimeter : FCSA ratio, while beyond 5000 µm^2^ there is barely any decrease in the ratio. In other words, the larger a fibre, the less perimeter per unit cross‐sectional area is available for capillaries. The perimeter : FCSA ratio allows us to calculate the maximum number of capillaries that can be packed on the periphery of a fibre with a given size. Here we assumed that a capillary has an external diameter of 8 µm (upper range of lumen in Kano et al., 2002) and hence we were able to calculate the theoretically maximal attainable CAF/FCSA for a fibre, assuming circular fibres, as illustrated by the black curve in Figure 2d. This curve will be even higher when the external diameter of a capillary is smaller, and/or when the fibre is not circular; then even more capillaries can be packed on the fibre perimeter, and hence a larger number of capillaries per unit fibre area (CAF/FCSA or CFD) can be achieved. An extreme case occurs when a fibre attains a flattened shape, seen in the European anchovy (*Engraulis encrasicolus*) where the perimeter : FCSA ratio is more than twice that of circular fibres (Johnston, 1982). However, no such adaptations have been seen in mammalian muscles during hypertrophy (Degens, 2012) nor in the highly resistance‐trained men in the present study (Figure 6b). The underestimation by assuming a circular circumference was relatively small, as reflected by an on average 14% larger perimeter than predicted from a circular circumference in muscle fibres from both mice and power lifters (Figure 6b). Nevertheless, the theoretically maximal‐attainable CAF/FCSA is far above that which is realised in the muscle cells, particularly for the largest muscle fibres.

One constraint that prevents full coverage of the perimeter of a fibre with capillaries is that during shortening the FCSA increases to a larger extent than the perimeter: for example, with 20% shortening of the fibre, the FCSA increases by 20%, while the perimeter increases by just 9%. The concomitant 20% shortening of the capillaries will result in increased capillary tortuosity (Mathieu‐Costello, 1989) which requires additional space on the perimeter of the fibre to accommodate the capillaries.

In addition to the physical constraint, the interstitial fluid has a low oxygen consumption, and therefore the interstitial $P_{O_{2}}$ gradient between capillaries around a given fibre is minimal (Honig et al., 1997), creating a high $P_{O_{2}}$ gradient from the interstitium to the muscle cell (Poole & Musch, 2023). Hence, any additional capillary on the periphery will result in a negligible, if any, impact on the oxygen flux from the periphery to the interior of the myofibre.

For another possible constraint for capillary supply to a fibre, consider a fibre with a radius (*r*) of 50 µm (diameter of 100 µm) and another with a radius of 25 µm. Assuming a circular circumference, these fibres will have an FCSA of 7854 µm^2^ and 1963 µm^2^, and a perimeter of 314 and 157 µm, allowing a maximum CAF of 314/8 = 39 and 20, respectively, as illustrated in the inset in Figure 2d. This results in a maximal CAF/FCSA of 5000 and 10,000 mm^−2^, respectively. The curves in Figure 6c show the increase in CAF/FCSA, or the capillary density, for a large (black line) and a small (grey line) fibre, with each additional capillary around the fibre. The red arrow shows that to attain a similar CAF/FCSA the large fibre must be surrounded by 4 times as many capillaries as the small fibre. This illustrates why small fibres have, when surrounded by the same number of capillaries, and hence in general, a larger number of capillaries per unit fibre area (CFD and CAF/FCSA) than large fibres.

Finally, Figure 6d illustrates that for each additional capillary on the periphery of a fibre, the reduction in supply area per capillary (and perimeter per capillary; data not shown) decreases. In other words, the benefit of adding a capillary around a fibre decreases exponentially, corresponding with diminishing returns with each additional capillary. Perhaps even more important is that no matter how many capillaries at the periphery of a fibre, the diffusion distance to the centre can never be less than the radius of the fibres as illustrated in the inset of Figure 6d. This will be particularly important for large fibres and applies not only to oxygen but also removal of metabolites and heat.

### 3.8 Diffusion and physical constraints of fibre size, oxidative capacity and capillarisation

**FIGURE 7:**
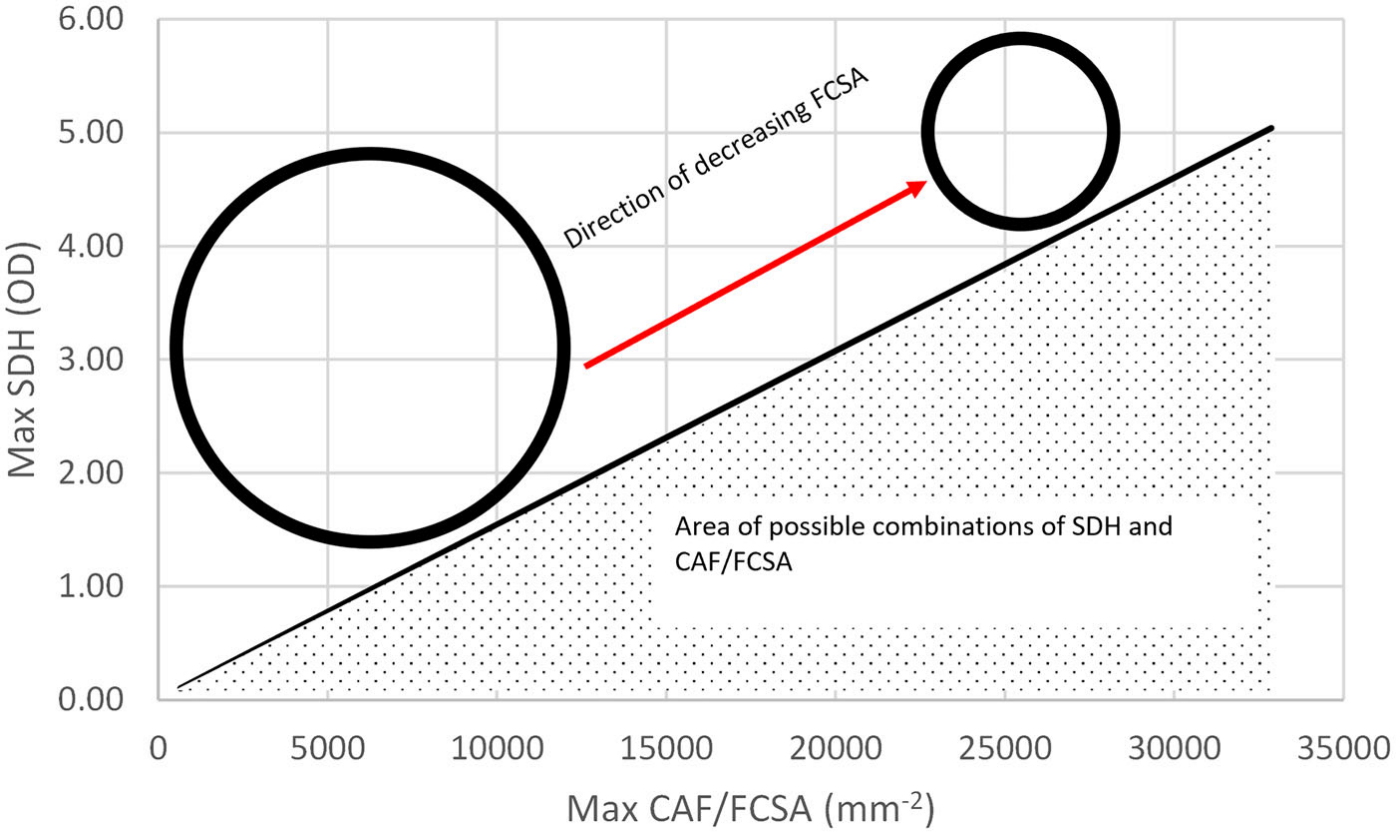
The relationship between the maximal succinate dehydrogenase (SDH) optical density, as a measure of fibre oxidative capacity, and the maximal number of capillaries around a fibre per fibre cross‐sectional area (CAF/FCSA) as calculated from the equations from Figures 2d and 5c, respectively.

In Figures 2d and 5c we fitted curves for the observed upper limit of CAF/FCSA versus FCSA and the upper limit of SDH OD versus FCSA, respectively. Using these equations, we calculated for each FCSA the maximal CAF/FCSA and maximal SDH OD. It appeared that the upper limits for SDH OD and CAF/FCSA were linearly related, where the FCSA decreased with increasing SDH OD and CAF/FCSA (Figure 7). While it has been suggested that the inverse relationship between SDH and FCSA is determined by diffusion constraints (Van der Laarse et al., 1998), the capillary supply to a fibre is not determined by diffusion constraints, but rather by physical constraints. That physical constraints limit the capillary supply to a fibre is suggested by: (i) no further decreases in maximal diffusion distance with more than two CAFs (see inset in Figure 6d), (ii) a diminished return for each additional capillary (in terms of the area or perimeter served per capillary) (Figure 6d), and (iii) physical limitation of space on the perimeter of a fibre (Figure 2d). It is interesting that the upper limits determined by physical constraints of capillary placement and the diffusion constraints for oxidative capacity are matched. Yet, there is significant room for adaptations in fibre size, oxidative capacity and capillary supply to a fibre as indicated by the dotted area in Figure 7. We hypothesise that a fibre will never have a combination of size, oxidative capacity and capillary supply in the white region of the graph, indicating a limit to muscular adaptations.

### 3.9 Escaping the diffusion and physical constraints

As adding more than two capillaries around a fibre does not reduce the maximal diffusion distance any more than the radius of the fibre (see inset in Figure 6d), the diffusion constraint is particularly problematic for large fibres, unless the fibre deviates significantly from a circular shape, such as the flat highly aerobic fibres of the European anchovy, which significantly shortens the diffusion distances from the capillary to the mitochondria (Johnston, 1982). We did not observe this in our study, nor has this been observed in hypertrophied mammalian muscle fibres. Another approach to reduce diffusion distances is fibre compartmentalisation with capillaries within – rather than only on the perimeter of – fibres as seen in the enormous fibres of the blue crab (Hardy et al., 2009). A few cases of muscle fibre splitting have been found in bird and rodent models of extreme loading (Murach et al., 2019), and human steroid‐using powerlifters (Eriksson et al., 2006). While this may be an adaptation to confer a biomechanical advantage in response to severe mechanical stress or a reflection of muscle fibre regeneration, it could also be an diffusional adaptation in instances when fibres become too large (Murach et al., 2019).

Another potential approach to break the size constraint is to improve oxygen diffusivity by (i) increased intracellular lipids that increase oxygen permeability as seen in cold‐acclimatised eel (*Anguila rostrata*) (Hoofd & Egginton, 1997), but whether this can help break the size constraint has not yet been investigated. Alternatively, (ii) myoglobin may facilitate oxygen diffusion, but it was not elevated despite a 40% fibre hypertrophy after ablation of synergists in rats (Masuda et al., 1997). In some cases, like in hypertrophied rat heart trabeculae, the oxidative capacity may not be supported by oxygen supply (van der Laarse et al., 2005), and after 4 weeks of exposure to hypobaric hypoxia there is an increase in the oxidative capacity of muscle fibres (Wüst et al., 2009). It was suggested that after exposure to hypobaric hypoxia the increased oxidative capacity may serve to maintain total mitochondrial respiration even though each individual mitochondrion may work submaximally (Hochachka et al., 1983).

Another possibility is the movement of mitochondria to the periphery of the fibre, thereby shortening the diffusion distances of oxygen from the capillary to the working mitochondria (Kinsey et al., 2007). While this does occur in the growing muscle fibres of the blue crab (Hardy et al., 2009), it causes problems with the diffusion of energy phosphates, such as ATP and P_i_ (Kinsey et al., 2011). It has been suggested that such a limitation of ATP diffusion is not a major problem for the ATP delivery to the myofibrils in the centre of the cell, as the proton gradient generated in the subsarcolemmal part of the mitochondrial reticulum can be used to generate ATP by the part of the mitochondrial reticulum in the core of the cell without the need of oxygen in the central mitochondria (Clanton, 2019; Glancy et al., 2015). This then may perhaps remove the oxygen diffusion constraints on fibre size and explain that (i) muscle fibre metabolism is typically not diffusion limited (Kinsey et al., 2011) and (ii) there is a dissociation between diffusion capacity and diffusion distances in immobilised dog muscle (Hepple et al., 2000).

We suggest, however, that the apparent independence of diffusion only applies when the size and aerobic capacity of the myofibres are within the range of possible combinations determined by diffusion and physical constraints (indicated in the dotted area of Figure 7). Indeed, in the animal kingdom, it has been seen that fibres are often on ‘the brink of substantial [diffusion] limitation’ and beyond a certain fibre size, muscle fibres undergo cell splitting or compartmentalisation (Kinsey et al., 2011). Previous studies suggesting breaking of the size constraint (Ballak et al., 2016; Hendrickse, Krusnauskas et al., 2021; Omairi et al., 2016) or showing a dissociation between diffusion capacity and diffusion distances (Hepple et al., 2000) were most likely adaptations to overload or immobilisation within the range of possible combinations indicated in the dotted region in Figure 7, as was the case for the muscle fibres from the highly resistance‐trained men in our study. It would be interesting to assess whether other models of hypertrophy in homeotherm vertebrates than highly resistance‐trained men obey or break the framework presented here.

As mentioned above, a mitochondrial ‘power grid’ that enables the transduction of the proton gradient from the paravascular mitochondria to the intermyofibrillar mitochondria (Clanton, 2019; Glancy et al., 2015; Parry et al., 2024) may break the diffusional size constraint for oxygen and ATP. The underlying assumption of the ‘power grid’ is that the proton gradient generated in paravascular mitochondria is, to all intends and purposes, instantaneously transferred to the mitochondria in the centre of the fibre. This requires the diffusion of protons (or the proto‐motive gradient/force) to be at least an order of magnitude faster than that of oxygen. Yet, the cytoplasmic diffusion coefficient for protons (diffusing as H_3_O^+^) of 9.3 × 10^3^ µm^2^/s (al‐Baldawi & Abercrombie, 1992) is almost three orders of magnitude less than that for oxygen of 2.4 × 10^6^ µm^2^/s (Fang et al., 2008) and at best only slightly higher than that for oxygen (2 × 10^3^ µm^2^/s) (Federspiel, 1986). In fact, the larger membrane potential in paravascular than intermyofibrillar mitochondria (Parry et al., 2024) – at least in resting muscle fibres – indicates that the mitochondrial potential is not transferred instantaneously from the periphery to the centre of the mitochondrial reticulum. Hence the elegant idea of a ‘power grid’ does at best only to a limited extent solve diffusion limitations.

Until now the focus has mainly been on the diffusion of oxygen, but the capillaries are also important for the delivery of substrates, and removal of heat and metabolites. Indeed, it has been suggested that the increase in capillary to fibre ratio in glycolytic fibres, without a change in their oxidative capacity, after chronic electrical stimulation indicated that capillaries were more important for metabolite removal than substrate (and oxygen) delivery (Egginton & Hudlicka, 2000). While this and the dissociation of diffusion capacity and diffusion distances after immobilisation (Hepple et al., 2000) indicate that there is no obligatory link between the oxidative capacity and capillary supply of a fibre – something we also observed previously (Bosutti et al., 2015) – we suggest that these observed adaptations occurred within the dotted area of Figure 7. In other words, these adaptations did not challenge the physical constraints of capillary placement around a fibre nor the diffusional constraints of oxidative capacity on the upper limits of attainable combinations of size, oxidative capacity and capillary supply to a fibre.

### 3.10 Conclusion

The inverse relationship between fibre size and maximal oxidative capacity for a fibre across muscles and species supports the notion of a trade‐off between oxidative capacity and size of a fibre, the ‘size constraint’ (Van der Laarse et al., 1998). The maximal capillary density of a fibre is limited by: (i) the maximum number of capillaries that can be placed at the periphery of a fibre, (ii) the diminished return of fibre volume serviced by a capillary with every addition of a capillary (Figure 6d), and (iii) the lack of any further reduction in the diffusion distance from the capillary to the core of the fibre by adding more than two capillaries on the periphery of a fibre. It thus appears that the oxidative capacity of a fibre is limited by diffusion constraints, while the maximal capillary supply to a fibre is limited by physical constraints. Based on our observations, our main conclusions are that (i) the maximal attainable size of a fibre is limited by its oxidative capacity, in accordance with the ‘size constraint’, and (ii) any increase in oxidative capacity of a fibre of a given size can only within limits be accommodated by concomitant angiogenesis.

## AUTHOR CONTRIBUTIONS

Hans Degens: conception and design of the work, and writing the first draft; Hans Degens, Guy A. M. Messa, Jason Tallis, Alessandra Bosutti, Tomas Venckunas, Ismail Adeniran, Rob C. I. Wüst, Paul W. Hendrickse: acquisition, analysis and interpretation of data, and revising it critically for important intellectual content. All authors have read and approved the final version of this manuscript and agree to be accountable for all aspects of the work in ensuring that questions related to the accuracy or integrity of any part of the work are appropriately investigated and resolved. All persons designated as authors qualify for authorship, and all those who qualify for authorship are listed.

## CONFLICT OF INTEREST

None of the authors have a conflict of interest.

## Data Availability

All data points are presented in the manuscript. Data can be accessed at: https://doi.org/10.23634/MMU.00637190. Further enquiries can be directed to the corresponding author.
